# Reliability of a New Indentometer Device for Measuring Myofascial Tissue Stiffness

**DOI:** 10.3390/jcm11175194

**Published:** 2022-09-01

**Authors:** Virginija Koch, Jan Wilke

**Affiliations:** 1 Diploma Hochschule, 37242 Bad Sooden-Allendorf, Germany; 2 Department of Movement Sciences, University of Klagenfurt, 9020 Klagenfurt am Wörthersee, Austria; 3 Institute of Occupational, Social and Environmental Medicine, Goethe University Frankfurt, 60590 Frankfurt am Main, Germany

**Keywords:** myofascial tissue, tissue stiffness, indentometry

## Abstract

Changes in tissue stiffness are associated with pathological conditions such as myofascial pain and increased risk of muscle injury. Furthermore, they have been shown to modify performance indicators such as running economy or jump height. Indentometry is an affordable way to assess tissue stiffness. However, to date, there is a paucity of studies examining the measurement properties of available devices. With this trial, we aimed to evaluate the reliability of the “IndentoPro”. Two investigators repeatedly measured the stiffness of the lateral head of the gastrocnemius muscle in healthy participants (N = 35), using 5 and 10 mm indentation depths. Intraclass Correlation Coefficients (ICC) revealed moderate inter-rater reliability (5 mm: ICC_3,1_ 0.74, 95%CI = 0.54 to 0.86, *p* < 0.001; 10 mm: ICC_3,1_ 0.59, 95%CI = 0.27 to 0.78, *p* < 0.001) and good intra-rater reliability (5 mm: ICC_3,1_ 0.84, 95%CI = 0.71 to 0.92, *p* < 0.001; 10 mm: ICC_3,1_ 0.83, 95%CI = 0.69 to 0.91, *p* < 0.001). No correlations between age, height, weight, BMI, skinfold thickness and myofascial tissue stiffness were observed (*p* > 0.5). In conclusion, the IndentoPro is reliable in assessing calf tissue stiffness, but the predictors of stiffness remain unclear.

## 1. Introduction

In recent years, soft tissue stiffness, the resistance of biological structures to an external deforming force [1,2,3,4,5], has attracted increasing interest among researchers and clinicians working with patients and athletes. This is mainly due to early evidence suggesting that the stiffness of the muscular and connective tissue may help identify pathological tissue states [1,2,6,7,8,9,10,11,12], monitor therapy or training effects [13,14,15,16,17,18] or prevent sports injury [19,20,21]. However, in the daily routine, most physiotherapists or trainers do not have access to sophisticated and complex measurement technologies (e.g., elastography) and thus, there is a need for straightforward and easy-to-handle devices capturing biomechanical soft tissue properties.

Indentometry is based on the standardized application of a compressive force deforming the underlying structures [22]. Using the amount of applied pressure and assessing the degree of indentation, it allows the calculation of tissue stiffness. A variety of handheld indentation devices [5,23,24,25,26,27,28,29] have been used for research purposes. All of them represent portable and relatively inexpensive means for measuring tissue mechanics in the clinical routine or during the training process. Validation studies have shown that indentometry devices are generally reliable in healthy individuals [5,24,25], but also in patients [23]. In an evaluation trial, Wilke et al. [5] examined the reliability and validity of a custom-made, handheld Semi-electronic Tissue Compliance Meter (STCM), concluding that this type of indentometry is an affordable, time- and cost-efficient method to evaluate the soft tissue properties of the lower leg. However, the device was not easily available to the public and hence, it is uncertain if commercial instruments may be able to reproduce the observed findings.

Building on the basis of said STCM [5], a new-generation device (IndentoPro, Technical University of Chemnitz, Germany and Fascia Research Project of Technical University of Munich, Germany) has been developed, which can measure soft tissue stiffness and elasticity with an indentometer function and the pressure–pain threshold with an algometer function at various indentation depths from 2 to 15 mm. However, to date, no study has examined the reliability of the IndentoPro even though it was already used in studies gauging the effect of interventions [30,31]. The present trial was therefore geared to elucidate its inter-rater and intra-rater reproducibility in assessing myofascial tissue stiffness.

Several definitions exist for the term “myofascial tissue”, with some authors emphasizing the importance of the intramuscular and intermuscular fascial tissue [12,32,33,34], while others weigh more the skeletal muscle [35,36,37,38,39,40] or both fascial and muscular tissue in conjunction [41]. In this study, we assume that the myofascial tissue is composed of the superficial fascia, deep fascia and the skeletal muscle with its connective tissue (e.g., endomysium, perimysium). The term myofascial tissue was also chosen because there is no ability to measure the stiffness of the muscle in complete isolation from other surrounding tissues when using indentometry. As the thickness of the subcutaneous connective tissue varies between different locations [42], it may influence indentometric measurements of myofascial stiffness. Besides investigating reliability, we therefore aimed to test whether correlations exist between skinfold thickness measured with a caliper and myofascial tissue stiffness measured with the IndentoPro, and/or between anthropometric data (gender, age, weight, height, BMI) and myofascial tissue stiffness.

## 2. Materials and Methods

### 2.1. Design and Ethics

A repeated measurements reliability study was prospectively registered in the German Registry of Clinical Trials (ID number DRKS00027417) and conducted in accordance with the Guidelines for Reporting Reliability and Agreement Studies (GGRAS) [43]. Briefly, using the IndentoPro, two investigators repeatedly assessed myofascial tissue stiffness of the calf at standardized intervals (see Section 2.3). Approval of the Ethics Committee of DIPLOMA University (approval number EB 1010/2021) was obtained. All procedures aligned with the principles of the Declaration of Helsinki and its recent modifications. Each of the participants provided written informed consent.

### 2.2. Participants

Healthy participants were recruited based on a convenience sampling technique [44]. The inclusion criteria were (1) willingness and (2) availability to participate in the. Exclusion criteria were based on those used by Wilke et al. [5], including trauma in lower extremity; surgery in lower extremity; orthopedic complaints and diseases in lower extremity; severe neurological, rheumatic, pulmonary or cardiovascular diseases; psychiatric disease; pregnancy; being in a nursing period; and painkiller intake in the past 48 h. Individuals were personally invited and recruited to take part in the study. Based on inclusion and exclusion criteria, N = 35 healthy subjects were recruited. Sample size selection was based on availability in this trial and hence did not represent the general population. However, the frequently recommended minimum of N > 30 was reached, which has been demonstrated to be sufficient to achieve statistical approximation to the standard normal distribution of the variables, and to achieve a validation of the study results [45,46].

### 2.3. Measurements

All measurements were conducted in a standardized manner and in constant environmental conditions (room temperature constant between 20° and 24° Celsius). Participants were examined in a closed investigation room, respecting the local COVID-19 pandemic regulations. All measurements, per participant, were carried out on the same day, in the dominant leg, and in the prone position. The dominance of the leg was determined by asking the question, “If you would shoot a ball on a target, which leg would you use to shoot the ball?”, as it is a reliable and fast method to determine leg dominance [47].

**Figure 1:**
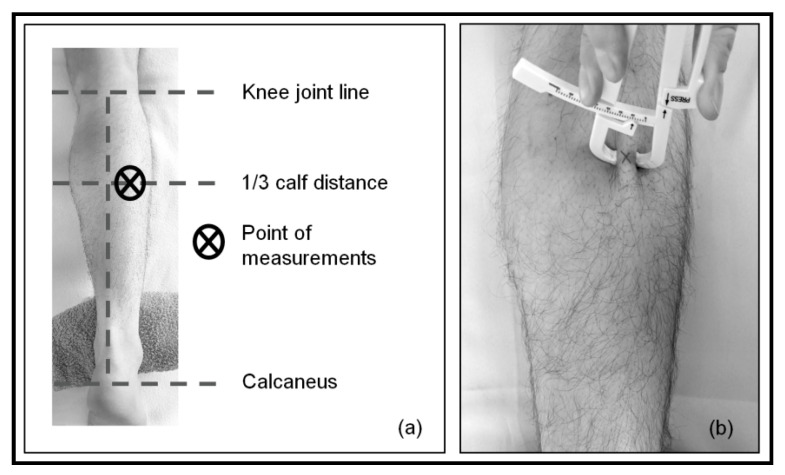
Location of the measurements. (**a**) Determination of the measurement location: cranial 1/3 calf distance, lateral head of gastrocnemius muscle; (**b**) measurement of skinfold thickness with skinfold caliper at the point of measurements.

Measurements were performed on the lateral head of the gastrocnemius muscle, at one-third (cranially) of the distance between the knee joint line and the calcaneus. To ensure consistency, the measurement location was marked with a water-soluble marker. First, skinfold thickness was measured once with the skinfold caliper (Acczilla Personal Body Fat Tester, Acczilla, Hong Kong, China; Figure 1).

Skinfold measurements were conducted by one examiner. After the skinfold at the site of measurement was firmly pinched between the thumb and the index finger of the investigator’s non-dominant hand, the jaws of the skinfold caliper (hold with dominant hand) were placed over the skinfold. Pressure on a press handle was applied with the thumb of the examiner’s dominant hand until the arrow of the press handle matched the arrow on the gauge, and subsequently, pressure was released. The thickness values shown on the scale were registered and used for further analysis.

**Figure 2:**
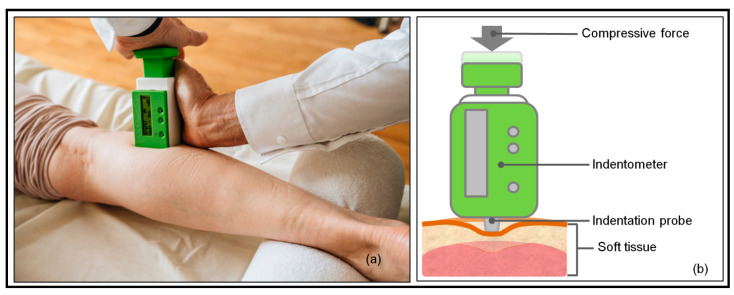
Myofascial tissue stiffness measurement with the IndentoPro. (**a**) Placement of the indentometer; (**b**) drawing of the stiffness measurement: after compressive force application, the indentation probe comes out of the indentometer body, indenting the soft tissue. The applied force (Newton) and resulting indentation depth (mm) are shown on a display.

Subsequently, myofascial tissue stiffness measurements were performed. Indentation depth (5 and 10 mm) was chosen based on the possible thickness of the skin and subcutaneous connective tissue in the calf region [48,49,50], indicating that the indentation depth of at least 5 mm should be used when measuring myofascial tissue stiffness. Although the device is able to measure the stiffness up to 15 mm indentation depth, measurements at 15 mm indentation depth were not included in the current study since pilot testing revealed inconvenient sensations (close to pain) at that depth. Two raters with professional physiotherapist qualifications carried out the myofascial tissue stiffness measurements as illustrated in Figure 2.

**Figure 3:**
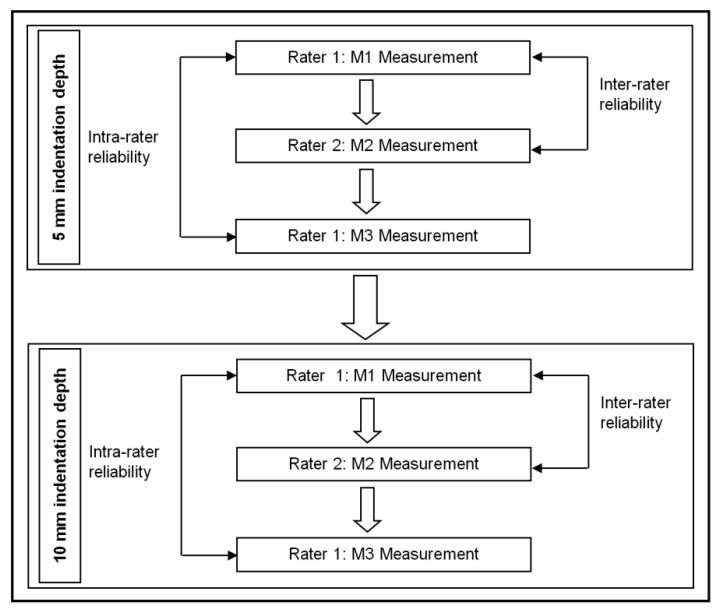
Chart displaying the workflow of reliability testing.

Three measurement sets were performed at both indentation depths (Figure 3). For each depth, the first rater performed the first (M1) and the last (M3) measurement set, while the second rater conducted the M2 measurements only. Each measurement set (M1 to M3) comprised 3 repetitions performed at 5 s intervals. Between the M1, M2 and M3 measurements, there was only a short break allowing for the change in raters. A 1 min break was used after all 5 mm indentation depth (M1, M2 and M3) measurements. Previous explorations confirmed that this amount of time between measurements was sufficient to avoid any noticeable viscoelastic adaptation processes (such as creep or hysteresis) due to repeated mechanical compression in this tissue region. Subsequently, the M1, M2 and M3 measurements were performed at a 10 mm indentation depth in the same sequence as the 5 mm indentation depth.

A maximal coefficient of variation of 15% (calculated and displayed by the device itself) between the trials of each measurement set (M1 to M3) was used as a cut-off score to determine a valid measurement based on the guidelines of the tool producer. No measurement had to be excluded during the study based on that cut-off score. Intra-rater reliability was determined by comparing M1 and M3 measurements at 5 mm and 10 mm indentation depth (Rater 1). Inter-rater reliability was examined comparing M1 (Rater 1) and M2 (Rater 2) measurements at 5 mm and 10 mm indentation depths.

### 2.4. Data Analysis and Statistics

Arithmetic means, medians, standard deviations (SD) and the range of variability (extreme values) were calculated for the quantitative variables. For qualitative variables, the relative frequency of their occurrence (percentage) was used. All quantitative variables were checked for normality using the Shapiro–Wilk test [51,52].

Comparisons of the results obtained in M1, M2 and M3 of the stiffness measurements were performed using non-parametric Friedman tests as an alternative for repeated measures analysis of variance (ANOVA) in order to identify potential systematic differences [45,52,53]. Lin’s Concordance Correlation Coefficient (Lin’s CCC) and Intraclass Correlation Coefficient (ICC) were calculated to assess inter-rater reliability and intra-rater reliability [52,54,55,56,57,58]. A two-way mixed ICC model with the definition of absolute agreement and single-rating score (ICC_3,1_) was applied [59,60].

Standard errors of measurement (SEM) were estimated to determine the absolute reliability using the formula “SEM = S_p_ × √(1-ICC)” [53]. The pooled standard deviation (S_p_ was calculated as “S_p_ = √(n_1_ − 1)s_1_^2^ + (n_2_ − 1)s_2_^2^/(n_1_ + n_2_ − 2)” [61]. Bland–Altman plots provided visual information on how widely scores deviated from the mean and the extent of agreement, expecting that 95% of the difference scores would fall within ±1.96 SD [52,53,62,63].

To report the quantitative strength of associations between the variables such as skinfold thickness and myofascial tissue stiffness, anthropometric data and tissue stiffness, Spearman tests were chosen [52,53,64]. The significance level of α = 0.05 was assumed for all comparisons. Statistical analyses were performed using IBM SPSS Statistics 28.0 (IBM Corp, Armonk, NY, USA) and NCSS 2021 (NCSS, LLC, Kaysville, UT, USA).

## 3. Results

### 3.1. Study Group Characteristics

In total, *n* = 35 healthy individuals (11 females and 24 males) took part in the study. Detailed characteristics of the sample are presented in Table 1.

### 3.2. Reliability

Descriptive data are shown in Table 2. Analyses of testing assumptions revealed non-normality of the data in some cases.

Since M1 and M3 measurements at 5 mm indentation and M2 measurements at 10 mm indentation showed a non-normal distribution (<0.05), Friedman tests were used to compare the measurements at 5 and 10 mm indentation depths. No significant differences (*p* < 0.001) were found between the different assessments of calf myofascial tissue stiffness, neither at 5 mm indentation (N = 35; Fr = 4.7; *p* = 0.95) nor 10 mm indentation depth (N = 35; Fr = 5.31; *p* = 0.70).

**Figure 4:**
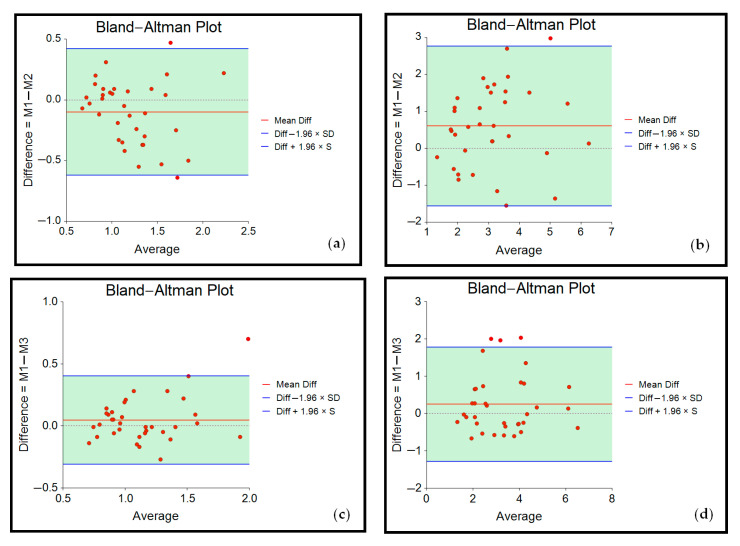
Bland–Altman plots showing agreement of measurements for (**a**) M1 and M2 at 5 mm indentation depth; (**b**) M1 and M2 at 10 mm indentation depth; (**c**) M1 and M3 at 5 mm indentation depth; (**d**) M1 and M3 at 10 mm indentation depth.

Bland–Altman plots depicting the respective measurements results are displayed below (Figure 4).

Bland–Altman plots for inter-rater reliability (M1/M2, mean difference scores: −0.10 at 5 mm, 0.61 at 10 mm) and intra-rater reliability (M1/M3, mean difference scores: 0.05 at 5 mm, 0.25 at 10 mm) showed that the points were scattered in an unbiased pattern, with most of them falling within the limits of agreement.

Lin’s CCC (0.58 to 0.74, Table 3) indicated moderate inter-rater reliability (M1/M2) and substantial intra-rater reliability (M1/M3, 0.82 to 0.84) for both indentation depths. ICC_3,1_ analyses (Table 3) revealed almost identical results.

### 3.3. Correlation between Skinfold Thickness, Anthropometric Data and Tissue Stiffness

Correlation analyses of anthropometric data (e.g., sex, age, weight, height, BMI) and myofascial tissue stiffness, in most cases, did not reveal significant associations (*p* > 0.05). Likewise, there was no correlation between skinfold thickness and myofascial tissue stiffness (*p* > 0.05, Table 4).

## 4. Discussion

The present study was the first to examine the reliability of the IndentoPro in the assessment of myofascial tissue stiffness. Our main finding is that the investigated device displays high reproducibility in repeated measurements by the same investigator and sufficient reproducibility when considering different investigators. With reference to intra-rater reliability, the results align closely with the previous study of Wilke et al. [5], who examined the previous generation of the IndentoPro. However, of note, contrarily to intra-rater reliability, inter-rater reliability values of the new device were slightly lower, which may be related to the increased complexity and the introduction of new functions. Notwithstanding, the IndentoPro can still be recommended for use in research and clinical practice. This is of significant clinical relevance, as this tool provides a time- and cost-efficient way of measuring myofascial tissue stiffness for practitioners in the field of musculoskeletal medicine within their daily clinical practice.

A second key observation of our study was that no association (only exception: M2 at 10 mm: weight) was found between tissue stiffness and person characteristics. This is in contrast to earlier studies reporting significant correlations between soft tissue stiffness and sex [18,65,66], age [17,65,66] or BMI [9]. Interestingly, the study by Wilke et al. [5] also failed to identify a relationship of stiffness and BMI or sex. Therefore, future studies further elucidating this issue may be of interest in order to clarify the role of potential factors moderating tissue stiffness.

Several studies [61,65,67,68,69,70,71] measuring skinfold or subcutaneous fat thickness with skinfold calipers or ultrasonography found negative correlations with tissue stiffness (mostly measured with the MyotonPro device [67,68,69]). Our study did not identify such associations. Besides being related to the assessment method, the lack of statistical significance in our study might be due to the small sample size and a consecutive lack of power.

Although no significant differences between the measurements at 5 mm and those at 10 mm were found, the data distribution at 10 mm depth seemed to indicate a trend towards a larger data variability. We suggest that this may be—at least partly—related to a larger variation in the manual pressure orchestration during the stronger tissue indentation process.

A variety of limitations must be recognized, and such limitations warrant future research. Firstly, we exclusively conducted stiffness measurements in the human calf region only. Expanding the focus to other body regions (i.e., trunk and upper limb) may be of value, particularly because this may allow direct comparisons to other studies using devices such as the Myotonometer^TM^ [23,24,25] and MyotonPro [26,27,28,29,72,73,74]. A second limitation relates to the indentation. Our data are novel in not only assessing stiffness at one depth. However, adding even more depths would allow the calculation of stiffness curves as a function of compression strength. Possibly, such dynamic evaluation would correlate more strongly with the above-mentioned potential predictors. In addition, different indentation depths might provide more specific information on different tissue layers such as the dermis, subcutaneous connective tissue, fascia profunda or muscular tissue. This hypothesis is based on the assumption that pressure needs to be higher if deeper structures are intended to be affected during stiffness measurements. Future investigations might therefore explore the suitability and reliability of different indentation depths for the assessment of different tissue layers. A third limitation relates to the fact that we recruited healthy participants only. It would be of interest to assess test properties in patients (e.g., with myofascial pain syndrome), as the focus of stiffness measurements is frequently therapeutic rather than preventative. Finally, despite the intriguing findings pointing towards sufficient to high reliability of the IndentoPro, a validation study is needed (e.g., measurement in patients with hypertension and asymptomatic persons or during different contraction levels) in order to verify that measured values are truly related to the construct of biomechanical stiffness.

## 5. Conclusions

This study provides evidence that the IndentoPro is a reliable device to measure myofascial tissue stiffness in the calf region of healthy individuals. However, as no systematic association was observed between stiffness and potential moderators such as skinfold thickness, sex, weight, height, BMI or age, further research is warranted in order to substantiate assumptions on the validity of the tool.

## Figures and Tables

**Table 1:** Sample characteristics and analysis of normal distribution.

Variable	Mean	SD	Med	Q1	Q3	*p*-Value (Shapiro–Wilk)
**Quantitative variable**						
Age, years	26.6	12.6	21.0	19.0	27.0	<0.001
Height, m	1.79	13.4	74.0	1.75	1.85	0.11
Weight, kg	74.8	0.09	1.79	65.0	85.0	0.68
BMI, kg/m^2^	23.3	2.8	23.4	21.1	24.7	0.55
Skinfold, mm	12.6	4.7	12.0	8.0	16.0	0.053
**Quantitative variable**		***n***			**%**	
Sex	Male	24			69	
	Female	11			31	
Dominant leg	Right	33			94	
	Left	2			6	

SD—standard deviation; Med—median; Q1—first quartile; Q3—third quartile; BMI—body mass index; *n*—number of participants.

**Table 2:** Descriptive statistics and analysis of normal distribution for myofascial tissue stiffness measurements.

Quantitative Variable		Mean	SD	Med	*p*-Value (Shapiro–Wilk)
**5 mm indentation**	M1, N/mm	1.16	0.36	1.09	0.004
	M2, N/mm	1.26	0.41	1.26	0.06
	M3, N/mm	1.12	0.30	1.16	0.01
**10 mm indentation**	M1, N/mm	3.40	1.38	3.22	0.16
	M2, N/mm	2.80	1.24	2.39	0.002
	M3, N/mm	3.16	1.36	3.05	0.10

SD—standard deviation; Med—median; M1—first measurement; M2—second measurement; M3—third measurement.

**Table 3:** Inter-rater and intra-rater reliability of the IndentoPro in measuring tissue stiffness.

Indentation Depth	M1/M2 (Inter-Rater)					M1/M3 (Intra-Rater)				
	Lin’s CCC (95% CI)	MSE	ICC_3,1_ (95% CI)	*p*-Value	SEM	Lin’s CCC (95% CI)	MSE	ICC_3,1_ (95% CI)	*p*-Value	SEM
**5 mm**	0.74 (0.55–0.85)	0.05	0.74 (0.54–0.86)	<0.001	0.20	0.84 (0.71–0.91)	0.03	0.84 (0.71–0.92)	<0.001	0.13
**10 mm**	0.58 (0.34–0.75)	1.14	0.59 (0.27–0.78)	<0.001	0.88	0.82 (0.68–0.91)	0.56	0.83 (0.69–0.91)	<0.001	0.56

M1—first measurement; M2—second measurement; M3—third measurement; CCC—Concordance Correlation Coefficient; CI—confidence interval; MSE—mean square error; ICC—Intraclass Correlation Coefficient; SEM—standard error of measurement.

**Table 4:** Correlations between anthropometric data, skinfold thickness and myofascial tissue stiffness.

Quantitative Variable	Indentation Depth	M1		M2		M3	
		r_s_	*p*-Value	r_s_	*p*-Value	r_s_	*p*-Value
**Weight**	5 mm	0.18	0.29	0.28	0.11	0.19	0.28
	10 mm	0.28	0.10	0.34	0.05 *	0.21	0.23
**Height**	5 mm	0.3	0.82	0.20	0.25	0.26	0.13
	10 mm	0.13	0.47	0.08	0.65	0.13	0.47
BMI	5 mm	0.12	0.51	0.30	0.09	0.17	0.34
	10 mm	0.29	0.09	0.44	0.009 *	0.17	0.32
**Age**	5 mm	0.03	0.86	0.08	0.64	0.04	0.80
	10 mm	0.06	0.77	0.08	0.63	−0.08	0.67
**Gender**	5 mm	0.22	0.21	0.31	0.75	0.21	0.24
	10 mm	0.26	0.13	0.20	0.24	0.29	0.09
**Skinfold thickness**	5 mm	−0.12	0.51	−0.11	0.52	−0.14	0.44
	10 mm	0.001	0.99	−0.04	0.83	−0.06	0.73

* Significant at the 0.05 level (2-tailed). M1—first measurement; M2—second measurement; M3—third measurement; r_s_—Spearman’s rank correlation coefficient.

## Data Availability

The datasets used and/or analyzed in the current study or any query regarding the research process are available from the corresponding author.
